# Applying Community-Based Participatory Research Partnership Principles to Public Health Practice-Based Research Networks

**DOI:** 10.1177/2158244016679211

**Published:** 2016-11-01

**Authors:** Nancy L. Winterbauer, Betty Bekemeier, Lisa VanRaemdonck, Anna G. Hoover

**Affiliations:** 1 East Carolina University, Greenville, NC, USA; 2 University of Washington, Seattle, USA; 3 Public Health Alliance of Colorado & Colorado Association of Local Public Health Officials, Denver, USA; 4 University of Kentucky, Lexington, USA

**Keywords:** practice-based research networks (PBRN), community-based participatory research (CBPR), academic–practice partnerships, communities of practice, knowledge co-production

## Abstract

With real-world relevance and translatability as important goals, applied methodological approaches have arisen along the participatory continuum that value context and empower stakeholders to partner actively with academics throughout the research process. Community-based participatory research (CBPR) provides the gold standard for equitable, partnered research in traditional communities. Practice-based research networks (PBRNs) also have developed, coalescing communities of practice and of academics to identify, study, and answer practice-relevant questions. To optimize PBRN potential for expanding scientific knowledge, while bridging divides across knowledge production, dissemination, and implementation, we elucidate how PBRN partnerships can be strengthened by applying CBPR principles to build and maintain research collaboratives that empower practice partners. Examining the applicability of CBPR partnership principles to public health (PH) PBRNs, we conclude that PH-PBRNs can serve as authentic, sustainable CBPR partnerships, ensuring the co-production of new knowledge, while also improving and expanding the implementation and impact of research findings in real-world settings.

## Introduction

As researchers across the social sciences dissect the ethical and moral implications of various approaches to fieldwork (de Laine, 2000; Zeni, 2001), participatory approaches have arisen that support collaborative decisions throughout the research process and resonate with participant values and perspectives (Anyaegbunam, Hoover, & Schwartz, 2010; Beltran, 1993; Brown, Howes, Hussein, Longley, & Swindell, 2002; Israel, Schulz, Parker, & Becker, 1998). While addressing ethical concerns and empowering non-academic participants, these approaches also provide vital ways of identifying real-world barriers and benefits for interventions (Graybill et al., 2010), addressing land-use and natural resource challenges (Ormsbee & Hoover, 2014; Smucker, Campbell, Olson, & Wangui, 2007), and optimizing the public health impact of research findings (Vanderpool, Brownson, Mays, Crosby, & Wyatt, 2013). Community-based participatory research (CBPR) provides a standard for partnered research that empowers non-academic participants in traditional communities (Krishnaswami, Martinson, Wakimoto, & Anglemeyer, 2012). Other field methods, however, also have developed that bring academic investigators together with practitioners from fields as diverse as medical care, education, and public health to identify, study, and answer questions relevant to these professions and their stakeholders. Public health practice-based research networks (PH-PBRNs) provide one example of such methods, partnering public health practitioners and academics to answer practice-relevant questions. As recognition grows of PH-PBRN capacity to produce practice-relevant findings (Mays, Hogg, Castellanos-Cruz, Hoover, & Fowler, 2013), the need to foster strong and lasting research partnerships has become evident. Adopting and adapting CBPR approaches could substantially advance the work of PH-PBRNs, particularly by refocusing partnership development and maintenance through an equity lens (Wallerstein, Duran, Minkler, & Foley, 2005).

### PH-PBRNs

The landmark 1988 Institute of Medicine report, *The Future of Public Health*, called for better integration of the public health academic and practice communities to improve public health practice (Institute of Medicine, 1988). The development and persistence of academic health departments (Erwin & Keck, 2014) and the emergence of our nation’s voluntary accreditation system for public health departments, which emphasizes evaluation and research (Public Health Accreditation Board & Accreditation Overview, 2013), provide evidence of some progress to this end. Aside from these examples, research evidence in the literature of the effective integration of the academic and practice communities in public health has been limited until recently. Driven largely by the growing need for efficacy in public health service delivery (Brownson, Fielding, & Maylahn, 2009; Kohatsu, Robinson, & Torner, 2004), the Robert Wood Johnson Foundation (RWJF) renewed interest in practice-academic research partnerships (Mays & Hogg, 2012). Since 2008, the RWJF’s promotion of and funding for PH-PBRNs has helped bring together public health academicians and practitioners to conduct practice-relevant research to increase the evidence base for effective public health systems (Mays et al., 2013). By 2012, PH-PBRNs existed in almost half of U.S. states, representing the involvement of more than 900 public health agencies and 35 academic institutions (Mays & Hogg, 2012).

PH-PBRNs partner academic researchers and public health practitioners to answer questions relevant to practice in the nascent field of public health services and systems research (PHSSR). PHSSR examines the “organization, financing, and delivery of public health services within communities, and the impact of these services on public health” (as cited in Mays, Halverson, & Scutchfield, 2003, p. 180). Examples of projects conducted by PH-PBRNs include the relationship of local health department expenditures to reductions in enteric disease (Bekemeier, Yip, Dunbar, Whitman, & Kwan-Gett, 2015); characteristics of local health departments with strong maternal and child health programs (Klaiman, Chainani, & Bekemeier, 2016; Klaiman, Pantazis, Chainani, & Bekemeier, 2016); increases in service delivery and other activities following the adoption of a core set of public health services in local health agencies (Lampe, Atherly, VanRaemdonck, Matthews, & Marshall, 2015); and factors affecting the adoption of evidence-based interventions in local health departments (Winterbauer, Bridger, Tucker, Rafferty, & Luo, 2015).

Network research has many advantages; by combining agencies it provides larger sample sizes, allows for comparative research across systems, is pragmatic, and results are readily translatable (Mays & Hogg, 2012; Mays et al., 2013). As such, PH-PBRNs are considered critical new translational links that can expand the scientific knowledge needed to improve public health practice and population health (Scutchfield, Mays, & Lurie, 2009).

### Clinical Predecessor

PH-PBRNs are modeled after clinically oriented PBRNs, which have focused on patient care and practice design in the United States for more than 30 years (Agency for Healthcare Research and Quality, 2012; Green & Hickner, 2006). Clinical PBRNs use physicians’ practical experience to define research questions relevant to practice improvement, address practitioners’ understudied research needs, and bridge the disconnect between research that is not easily translated to medical practice (Mold & Peterson, 2005; Nutting, Beasley, & Werner, 1999). To improve research relevance and translation, clinical PBRNs also involve providers in research as end users (Green & Hickner, 2006; Nutting et al., 1999). In clinical PBRN partnerships, clinicians and researchers work together to examine practice characteristics with the short-term goal of practice improvement and the long-term goal of improved patient health.

### CBPR and PH-PBRNs

Participatory or community-engaged research (CEnR), which promotes research relevant to and actionable in communities, has a long history, particularly in education, the social sciences (Wallerstein & Duran, 2008), and public health (Faridi, Grunbaum, Gray, Franks, & Simoes, 2007; Wallerstein & Duran, 2010). The degree of community or lay research participation exists along an engagement continuum, in which control of the process is anchored on one end by researchers and on the other by community participants (Cornwall & Jewkes, 1995; Lesser & Oscós-Sánchez, 2007). The term CBPR captures the twin ideals of action-oriented and community-partnered research (Minkler & Wallerstein, 2008). With a focus on the equitable engagement of lay researchers throughout the research process and increasingly in policy research, CBPR aligns well with the principles of social justice and human rights embodied in public health’s attention to the social determinants of health and health equity (Cacari-Stone, Wallerstein, Garcia, & Minkler, 2014; Israel et al., 1998; Wallerstein & Duran 2010).

While CBPR has been variously defined, descriptions often focus on the WK Kellogg Foundation (2013) characterization of CBPR as

a collaborative approach to research that equitably involves all partners in the research process and recognizes the unique strengths that each brings. CBPR begins with a research topic of importance to the community and has the aim of combining knowledge with action and achieving social change to improve health outcomes and eliminate health disparities.

CBPR builds bridges between scientists and communities by involving community participants and researchers in all aspects of the research beginning with identifying the issue to be addressed, research design, implementation, and dissemination. It has been found to enhance the relevance, quality, and use of research data by increasing the likelihood of overcoming distrust of research by communities that have traditionally been only the “subjects” (Israel et al., 1998; Israel, Schulz, et al., 2001). It benefits practitioners, researchers, and participants through shared knowledge and experiences that result in more relevant research questions and more effective interventions (Israel et al., 1998; Viswanathan et al., 2004). However, a CBPR approach takes time, as relationship building takes time and consensus decision making is the preferred method of coming to agreement (Israel, Schulz, et al., 2001; Viswanathan et al., 2004). Despite these challenges, CBPR offers a means to reduce the gap between theory, research, and practice (Israel et al., 1998). Consequently, CBPR is considered the gold standard for participatory research because its principles directly, collaboratively, and iteratively address power imbalances across the community-engaged spectrum (Wallerstein et al., 2005).

Because PH-PBRNs are intended to conform to participatory ideals (Mays & Hogg, 2012), similarities exist between the development of evidence through PH-PBRNs and CBPR approaches. Both call for relationship building and maintenance, draw on insider knowledge to articulate practical research questions, require non-academic partners to participate in research processes, and rely on diverse partners to translate new knowledge into action (Wallerstein & Duran, 2010).

As with other forms of participatory research, however, academics and practitioners engaged in PH-PBRNs encounter long-standing collaboration challenges that foster mistrust. These challenges include community members’ and practice partners’ limited research experience, resource constraints related to time and funding, historical failure to compensate communities and practitioners, and negative attitudes about the real-world relevance of research (Befort, Orr, Davis, Ely, & Steiger, 2009). As has been the case in many traditional communities, practitioners have characterized academics as inaccessible, devaluing non-academic skills and knowledge, and being unfamiliar with real-life demands (Befort et al., 2009), including the frequent need to make practical decisions without sufficient data. As with many traditional community collaborators in CBPR partnerships, PH-PBRN practitioners also are often disadvantaged in the research context, with academics typically controlling resources, methods, and dissemination of results (Winterbauer & Myers, 2013).

Like clinical PBRNs, practitioners are the end users of evidence generated through PH-PBRNs, as research results are intended to improve practice in the short-term and contribute to the long-term goal of achieving healthier communities. This is a key distinction between traditional CBPR partnerships and PBRN partnerships.

Thus, in the paragraphs that follow, we posit that the “community,” in PH-PBRNs, is the public health practitioner community and consider the applicability of CBPR partnership principles to PH-PBRNs. Specifically, we evaluate whether and how CBPR principles could fit within the PH-PBRN context, one that includes practitioners as the “community of identity” (Israel et al., 2003, p. 55), and contribute conceptually and operationally to PH-PBRN development and sustainability, as a model for PH-PBRN partnerships.

## Process

Using the nominal group technique (Cantrill, Sibbald, & Beutow, 1996), the authors formed an expert panel to generate and explore ideas regarding the applicability of CBPR guidelines in PH-PBRN development and maintenance. Because our topic addressed both PH-PBRN development and CBPR, the panel comprised individuals with expertise spanning both domains. Our panel of four included three founding members of three different statewide PH-PBRNs originally funded by RWJF, and thus among the most mature of the PH-PBRNs, and one with experience supporting the development of PH-PBRNs nationally.

Three members of the panel currently have full-time academic positions and one has a practice position. Two are “pracademics” (Mays & Scutchfield, 2012) with experience in academic public health and public health practice. Together, the panel has close to 30 years of public health practice experience and 45 years of academic experience. Our PHSSR interests include public health finance, communication, core services, public health and primary care partnerships, public health and hospital partnerships, shared services among local public health agencies, quality improvement, and accreditation. Two of us have studied CBPR tenets in-depth and have embraced them in our own research in areas as diverse as service delivery models for special needs children, criminal justice, substance abuse, and mental health diversion programs, childhood obesity, environmental health, community environmental decision making, and preparedness-related risk communication.

For this inquiry, we focused on Israel and colleagues’ (2003) nine CBPR principles (Table 1). Meeting regularly by telephone, our panel discussed the applicability of each CBPR principle to the dynamics of PH-PBRNs. Each session focused on one principle, with some principles requiring multiple sessions. Panelists independently reviewed the focus principle prior to each session, formulating individual reflections informed by literature and their own experiences with CBPR and PH-PBRN partnerships. During the regular discussion sessions, we shared insights, contested interpretations, synthesized perceptions, discussed literature, and achieved convergent understandings of specific relationships between each principle and PH-PBRN research. We recorded discussions, and the lead author maintained rigorous notes, with the panel subsequently reviewing each discussion to achieve agreement. Upon achieving convergence about the applicability and implications of each principle, the panelists conducted presentations and listening sessions at both the National Association of County & City Health Officials (NACCHO) and the Keeneland Conference on PHSSR to conduct member checks, sharing preliminary conclusions and garnering additional insights and alternative perspectives from the wider PH-PBRN community before reaching the final conclusions outlined in this manuscript.

## Findings

The panel reached consensus regarding each CBPR principle (Israel et al., 2003) and its adaptiveness to the PH-PBRN framework as summarized in Table 1 and described below.

### 1. CBPR recognizes community as a unit of identity

*Community* is central to CBPR and is defined as a shared sense of identity (“communities of identity”) that binds members through common interests and a “commitment to meeting shared needs” (as cited in Israel et al., 2003, p. 55). While traditional CBPR communities may be composed of clients, neighborhood residents, or issue-focused coalitions, PH-PBRNs partner state and local public health practitioners who share insider knowledge of real-world practice and interests in producing an evidence base for public health with researchers who share these interests and have the training, resources, and skills for conducting research.

PH-PBRN membership has generally included only academics and practitioners. For example, the New Jersey Public Health PBRN is led by the New Jersey Department of Health, a state office, in partnership with the New Jersey Medical School (NJMS) and the School of Public Health (SPH) of Rutgers University. The network team also includes the New Jersey Association of County and City Health Officials; the Rutgers Office of Continuing Professional Education; and Region 2 Public Health Training Center (PHSSR & PH-PBRN, 2016b). Similarly, the Washington PH-PBRN is led by the Public Health-Seattle & King County local health department and includes local health departments serving the nine largest jurisdictions within the state, along with the Washington State Department of Health, the Washington State Association of Local Public Health Officials, and research partners at the University of Washington School of Public Health and Community Medicine and School of Nursing (PHSSR & PH-PBRN, 2016c).

As such, PH-PBRNs vary from more established CBPR community partnerships by focusing on partnerships with service providers, who might be considered privileged and, therefore, inauthentic in comparison with more traditional CBPR arrangements (Tapp & Dunlin, 2010). In business and in health care, however, partnering across service sectors has been described as the formation of *communities of practice* (Li et al., 2009). These formal and informal professional networks—often spanning professions, organizations, and agencies—are learning communities that support evidence-based practices to optimize system performance. The communities of practice undergirding PH-PBRNs share a commitment to better system performance that can contribute to improved community health status.

We postulate that *the “community” first impacted by results from PH-PBRN studies is the practice community of public health providers and administrators*, whether through policy change or through enhanced administrative, delivery system, or service practices. The client communities served by public health agencies are affected indirectly by PH-PBRN research, through improved public health practice. We believe that recognizing the communities of practice comprising PH-PBRN partnerships as authentic communities of identity provides a useful platform through which academics and practitioners can collaborate to build evidence for improving public health practice and as such, position them to benefit from the principles of practice articulated by the CBPR community.

### 2. CBPR involves systems development through a cyclical and iterative process

Israel and colleagues (2003) referred to partnerships as systems that develop capacity to sustain themselves, grow, and engage in research. Productive CBPR partnerships require explicit attention to partnership development and sustainability. This gains salience as CBPR researchers and the communities they work with often inhabit markedly different economic and sociocultural milieus, leading to tension and mistrust (Israel et al., 1998; Wallerstein et al., 2005)

Academics and public health practitioners also inhabit different cultural worlds (Winterbauer & Myers, 2013), which can create similar partnership development and sustainability challenges. Our panel came to consensus that, like CBPR partnerships, *strong PH-PBRN partnerships require trust-building through deliberate and bidirectional attention to understanding each partner’s values and professional environments* (Winterbauer & Myers, 2013). This includes valuing differences in system-level rewards and penalties, motivators, scopes of interest, and work parameters among all partners.

### 3. CBPR facilitates collaborative, equitable partnership in all phases of the research

Equitably shared power over decision making is considered among the “most critical” CBPR elements (Israel et al., 2003). Intended to imply fairness rather than sameness, equity is highly contextual and must be defined locally by each partnership (Israel et al., 2003). In traditional CBPR partnerships, power imbalances may appear obvious when research collaboratives include disenfranchised or under-resourced populations. Although perhaps less obvious, power imbalances also are inherent to PH-PBRN collaboratives. We identify three explicit areas in which imbalances can negatively affect PH-PBRN development and sustainability, potentially reducing research relevance and rigor and minimizing translational impact: (a) control of the research process, (b) resource sharing, and (c) dissemination of results.

#### Control of the Research Process

Whoever makes decisions during the research process controls the direction and consequently, outcomes of the research itself. Collaborative decision-making processes are tied to each partner’s expertise, interest, and desired role, which varies among PH-PBRN practice partners who sometimes have little or no formal research training and for whom research is not the first priority. Such variance in both expertise and interest can substantially affect the expectations of all network partners, as illustrated by an exchange described by one of the authors. In this instance, a local public health director who was co-principal investigator of a PH-PBRN study remarked while reviewing results from preliminary analysis, “We thought we would give you [academic partners] the question, and you would give us the answer.” Consequently, to ensure equity and maximize contributions of practice-focused partners, our panel resolves that *explicitly defining roles and expectations is a critical ongoing step* in practice-academic research and that the burden for ensuring this falls to stakeholders for whom research is a priority—often the academic partners.

#### Resource Sharing

In PH-PBRN projects, practitioner partners sometimes contribute to knowledge production in understated but critical ways. For example, practitioner partners assist in recruitment; collect and/or provide access to data; contribute to analysis and data interpretation; and translate and communicate results among colleagues taking responsive action. The time and resources invested in these activities can be overlooked and, consequently, not compensated. Uncompensated investment implies inequity and is noticed by our practice partners. *Documenting effort and effective role definition* can contribute to equitable compensation and resource sharing, thereby building trust toward and enabling improved co-production of knowledge.

#### Results Dissemination

Control of knowledge dissemination is a form of power. Early dissemination and translation of research results are cornerstones of both CBPR (Wallerstein & Duran, 2010) and PH-PBRNs (Mays et al., 2013). Rapid uptake is expected when the research questions and results are derived from and are meaningful to the community, here, the community of public health practitioners. In PH-PBRNs, practitioner partners have both the interest and the authority to act on results; therefore, *dissemination plans should feature practitioners actively guiding early release of results to optimize real-world uptake and impact.* Venues meaningful to the public health community and to which practice partners often are gatekeepers include local health directors and boards of health meetings, as well as state and national public health association meetings. Academics can support preparation of translational research briefs for practice audiences, while taking the lead in disseminating findings to research audiences via disciplinary conferences and manuscripts, thereby meeting the needs of their own professional incentive systems.

### 4. CBPR integrates and achieves a balance between research and action for the mutual benefit of all partners

CBPR generates knowledge to create *social* change (Israel et al., 1998), while PH-PBRNs generate knowledge to stimulate *organizational* change. Our panel considered two PH-PBRN challenges to this principle that mirror CBPR partnership challenges: defining mutual benefit and determining scope of inquiry (Wallerstein et al., 2005).

Defining mutual benefit can be challenging when partners are embedded in very different career tracks, organizational cultures, and sociocultural milieus. While both PH-PBRN research and practice communities share the goal of improving public health systems and community health, these communities of practice differ in the scope and nature of their work. Practitioners are both professionally and personally rewarded when best practices and policy change improve community health, while the career advancement of academic researchers is tied directly to a tenure system that incentivizes the generation of grant funding and peer-reviewed publications. These differences can create tensions that affect the quality of research and effectiveness of dissemination and translation efforts when practice concerns related to feasibility, timeliness, and actionability conflict with academic concerns about research rigor and publication production.

Moreover, relationships can become stressed when, for example, the political environment constrains research that could produce results unfavorable to a public health program, but that would advance the professional success of an academic investigator (Minkler et al., n.d.). Similarly, because the unit of analysis in many PHSSR studies is the public health jurisdiction (e.g., county), PH-PBRN study results can jeopardize the standing of local agencies, which vary in governance structure and level of autonomy (Association of State and Territorial Health Officials [ASTHO], 2014; Centers for Disease Control and Prevention, 2016; Hyde & Shortell, 2012). Local health departments are often answerable to multiple constituencies, which may include oversight bodies, such as state health departments, local boards of health, and county commissioners, as well as diverse community partners, each with their own power, interests, and agenda (Mays & Scutchfield 2010; Winterbauer et al., 2015). Consequently, it is paramount that the privacy and confidentiality of health information assured individual research participants be extended to protect the confidentiality of organizations, which may be challenging.

For example, in North Carolina, as in many states, results presented by population size of jurisdiction could easily identify the few large jurisdictions in the state. In these cases, PH-PBRN academic partners must work closely with their practice partners to identify if or how results will be disseminated. We conclude that *attention to the drivers and limitations of partner groups must be respected through ongoing dialogue* as members choose, conduct and report research that yields action.

### 5. CBPR promotes co-learning and capacity-building among all partners

Fundamental to participatory research is that, working together, academics and community members have the skills and resources needed to conduct rigorous, practically meaningful research (Israel et al., 1998). Recognizing, valuing, and sharing these skills and resources underlies the promise of CBPR. Co-learning suggests mutual exchange of skills and ideas through reciprocal transfer of knowledge, while capacity-building suggests that increased access to resources and expertise can improve research outcomes and optimize real-world impact. Our PH-PBRN experiences with co-learning indicate that academic researchers share expertise in research design and analysis, while practitioners contribute tangible expertise such as elucidating the coding nuances within data sets and providing key insights about variations in service delivery that might affect study design or interpretation of results.

An example from the public health finance literature illustrates how the process of practice-academic co-learning can enhance the rigor of practice-based research and hence the value of these collaborations. Recent work in PHSSR has emphasized the need to determine the cost of providing public health services (Honore, 2015; Institute of Medicine, 2012). However, public health practitioners across the country are unlikely to have the empirically-based cost data necessary to make such determinations (Budetti & Lapolla, 2008). The NC PH-PBRN worked with environmental health directors and finance managers in local health departments across the state to develop a tool to estimate the cost of delivering environmental health services (Winterbauer, Singh, Tucker, & Harrison, 2016). The tool was based on one developed to estimate the cost of substance abuse services (Zarkin, Dunlap, & Homsi, 2004) and was designed to estimate the full costs of two categories of environmental health service lines: food and lodging inspections and onsite water services (Singh, Winterbauer, Tucker, & Harrison, 2015, in press).

In formative research with practice partners, several challenges to valid cost estimates emerged. These were primarily related to agencies not tracking direct labor and non-labor costs by program area, inexperience with indirect or overhead costs, and the county government absorbing some agency costs, for example, rent (Winterbauer et al., 2016). Intense practitioner participation was crucial to establishing valid measures during tool development. Similarly, practitioners and researchers worked collectively during the formative period to balance measurement rigor or reliability, with practice constraints, by defining measures that would provide reasonable cost estimates without placing unreasonable demands on health department staff responsible for data collection.

Opportunities for reciprocal capacity-building in PH-PBRNs have not been well described in the literature. From our own PBRN experiences, our panelists identified several concrete capacity-building examples that have proven useful. For example, in support of practice partners, local health department staff have been provided electronic access to academic libraries, accompanied by online training in literature searches and a webinar on creating professional posters. Face-to-face training has included results-based accountability and panel presentations have described lessons learned in local health department—hospital partnerships to conduct community health assessments. One of our PH-PBRNs hosts scientific sessions at the annual state public health association meeting and explicitly encourages practitioner (and student) participation by offering awards for best presentations. Other broad practitioner training we believe would be useful includes training in Institutional Review Board scope and process, general research design, and participatory research. Academics typically benefit from these partnerships by gaining access to health department databases, but may also benefit through inclusion on committees and task forces, which can broadly strengthen understanding of emerging and current public health issues.

Emphasizing the reciprocal nature of co-learning and capacity-building in this context, each stakeholder group both gives and receives. We conclude that, where possible, *academics and practitioners should identify and institutionalize opportunities for reciprocal exchange of ideas, skills, and resources* that enhance their collaboration. We also recognize these exchanges as exemplars of an ongoing commitment to partnership that not only enhances the probability of success in current research activities but also potentiates the joint pursuit of future research and translational opportunities.

### 6. CBPR builds on strengths and resources within the community

Highlighting the necessity of diverse participation in participatory research, Israel and colleagues (2003) noted that expanding and supporting partnerships to build community capacity is an important CBPR objective. This perspective is borne out by evidence from PH-PBRNs indicating that public health agencies participating in PH-PBRNs are engaged in research at far higher levels than non-participating agencies. However, great variation exists in the membership, organizational structures, and interaction patterns across PH-PBRNs (Mays & Hogg, 2012), with social network analysis revealing that local public health practitioners tend to be less engaged and perceive fewer benefits from network participation than academics (Mays et al., 2013). Because organizational sensemaking capacity is constrained by the number and diversity of available actors (Weick, 1988), we conclude that both the quality and volume of practice-relevant PH-PBRN research are similarly constrained by the collective capacity of the network’s members.

Multisectoral partnerships, which emphasize interprofessional and cross-sector collaborations, are at the core of effective public health practice and considered critical to achieving population health goals, including health equity (Taillepierre et al., 2016; Teutsch & Fielding, 2013; Woulfe, Oliver, Zahner, & Siemering, 2010). As PH-PBRNs evolve, *networks should actively recruit members from both traditional public health organizations and other sectors*, including community health centers and community-based organizations, to incorporate a greater diversity of strengths and resources from within their communities. By providing a wider lens through more diverse participation, PH-PBRNs can create a stronger foundation for identifying research questions, designing and conducting research, and increasing translational impact.

### 7. CBPR emphasizes local relevance of public health problems and ecological perspectives that recognize and attend to the multiple determinants of health and disease

PH-PBRNs are intended to be practice-driven and locally relevant, conducting research focused on the broad public health system and its organizational services and structures (Mays & Hogg, 2012). As such, PH-PBRN research focuses on the outer rings of the ecological model: policy, organization, and environment (system), with relevance to local practice (McLeroy, Bibeau, Steckler, & Glanz, 1988). Studies conducted by PH-PBRNs are intended to have implications for improving health from an “upstream” social determinants of health perspective, as they are focused on improving policies and systems to enhance a community’s health.

The focus on practice relevance presents two challenges to the work of PH-PBRNs: generalizability of research results and funding availability. Practice relevance is critical to attracting and maintaining practice partner interest, but can limit the generalizability of results. In comparison with case studies, the network structure of PH-PBRNs is intended to increase sample sizes by including all public health jurisdictions within a state in the network. However, states vary in the number of jurisdictions they contain (range 2-329, Delaware and Massachusetts, respectively; National Association of County and City Health Officials [NACCHO], 2014) and there is a great deal of variation at the local level within states, as captured by the adage, “If you’ve seen one local health department, you’ve see one local health department.” Moreover, the complexities of research questions may limit analyses, even in states with a moderate number of jurisdictions, to relatively simple statistics (e.g., Singh et al., in press). In these cases, descriptions of the research context become especially important in considering generalizability. Otherwise, where designs allow, states may carefully pool samples (e.g., Bekemeier et al., 2015). It should be noted that PHSSR is a relatively young area of study and PH-PBRN partners are working collaboratively to define the field, including standardized measures for comparative purposes (Public Health Activities and Services Trackin, n.d.; PHSSR & PH-PBRN, 2016a).

Similarly, although academic and practice PH-PBRN partners participated in constructing the PHSSR national research agenda that established funding priorities (Consortium from Altarum Institute, 2012), funding from federal and philanthropic organizations may not be timely or fit current practice needs or interests specific to PH-PBRNs, which are likely to be changeable. Further, human resources capacity to pursue specific studies may be lacking. For example, an investigator with expertise in an emerging, practice-relevant, system-level problem that a PH-PBRN wishes to study might be unavailable. Our panel agreed that addressing locally relevant research questions through an ecological, upstream perspective is inherent in the PH-PBRN ethos; however, funding and resources of necessity drive what is researched. Consequently, *networks must* act *strategically* to *develop collaborative, practitioner-driven research programs that can evolve and thrive in the face of funding and resource realities*.

### 8. CBPR disseminates findings and knowledge gained to all partners and involves all partners in the dissemination process

CBPR produces actionable knowledge (Israel et al., 1998), while PH-PBRNs also produce results intended to address public health problems (Mays & Scutchfield, 2012). Thus, translation is central to the research enterprise in both settings. Israel and colleagues (2003) have recognized important role- and power-related questions involved in dissemination of results, including how results are disseminated, who the “voice(s)” of dissemination is, and how authorship is determined. Our panel agrees that *research collaboratives must address dissemination roles*, particularly as they relate to variations in the values and reward systems of network partners.

In PH-PBRNs, the “lay” audience is the public health practice community. Thus, the contextual and professional knowledge of practice partners, as well as their recognized roles in the larger practice community, positions them as key gatekeepers who can legitimize research to practitioners in ways that academic partners often cannot, and vice versa. As a result, practitioners often disseminate findings via presentations and structured dialogue at association conferences and in other professional venues, such as statewide health director and state-specific public health association meetings. PH-PBRN practice partners are uniquely situated as change agents to disseminate and promote translation among their peers (Winterbauer et al., 2015).

Academics similarly disseminate findings through disciplinary conferences and the requisite peer-reviewed publications. We recommend that PH-PBRNs *pay explicit attention to the differences in dissemination opportunities, experiences, and expertise among network members* to ensure results are successfully shared with relevant audiences for mutual benefit and optimal impact. Unconventional approaches, such as having academic researchers attend practice-focused meetings and practitioners present in academic environments, also may help achieve this goal while building partner capacity for understanding each other’s perspectives. Additionally, national organizations such as the National Association of County and City Health Officials (NACCHO) and AcademyHealth frequently highlight PHSSR and PH-PBRN research in their conferences, encouraging both academic and practice audiences to attend and present research.

### 9. CBPR involves a long-term process and commitment

The historically poor relationships between academics and the communities they study may be partially due to the traditional “drive-by research” model in which academics engage in partnerships until their professional needs are met, then deserting communities upon completion of a research project (Horowitz, Robinson, & Seifer, 2009). In our panel’s experiences conducting and coordinating PH-PBRN research, we have heard this sentiment from public health practitioners and experienced the consequences of these previous negative encounters between practice and academia. Partnership sustainability, particularly when resources are scarce, is challenging. This is no less true for PH-PBRN partnerships.

The 2008 creation of the first PH-PBRNs in response to an RWJF call for proposals encouraged governmental public health practice settings as lead network partners (National Coordinating Center for PHSSR, 2008); however, by 2012, approximately just less than one third of all PH-PBRNs registered with the National Coordinating Center were led by state public health associations and a bit more than a third by academic institutions; the remaining 31% identified state or local public health agencies as lead partners, with only roughly one fifth of these led by local agencies (Mays & Hogg, 2012). This shift underscores the challenge of developing and sustaining PH-PBRNs grounded in practice agencies that must meet community public health needs daily and, therefore, necessarily recognize research activity as a lower priority.

Nonetheless, while public health practice and academic communities occupy different worlds, professional circles do overlap, which allows for significant interaction and commitment to one another outside PH-PBRN research-related activities. We conclude that such *routine interactions as student internships, evaluation projects, and/or joint participation on statewide committees provide a foundation for partnership development and maintenance*, even in the absence of research funding, thereby demonstrating ongoing commitments to long-term partnership.

## Discussion

PH-PBRNs have demonstrated that they can produce practice-relevant research and translate that research for real-world impact in practice settings (Mays & Hogg, 2012; Mays et al., 2013). Like CBPR partnerships, PH-PBRNs help “bridge the gap between science and practice” (Wallerstein & Duran, 2010), providing powerful opportunities to produce evidence that enhances practice (Vanderpool et al., 2013; Wallerstein & Duran, 2010). However, like other multisectoral partnerships, these research collaborations are challenging.

The aim of this inquiry was to determine how CBPR partnership principles might enhance the development and sustainability of PH-PBRNs, thereby improving both research rigor and real-world relevance. We strongly believe that the CBPR emphasis on the partnership process provides a roadmap through which academic- and practitioner partners can negotiate the power dynamics that inform every step of the research process—from identification of research questions, through research design and implementation, and the dissemination and translation of study findings for real-world public health impact.

CBPR principles also highlight the importance of developing trust through power- and resource sharing, explicit negotiation of research responsibilities, and attention to partners’ cultural and political milieus. Power imbalances can undermine the development and maintenance of trusting environments necessary for research collaboration. However, formal role negotiation, although rare in our PH-PBRN experiences to date, can facilitate such collaboration. Several strategies can be used to acknowledge expertise, responsibilities, and decision-making authority of practitioner and academic partners, including acknowledging where and how team members will participate, designating who will lead each study phase, and indicating the level of effort expected from each member. Such strategies can reduce ambiguity, streamline research processes, solidify relationships, provide a basis for determining compensation, and enhance both capacity and opportunities for effective dissemination.

Examining PH-PBRNs through this lens of CBPR principles foregrounds the variation in cultural and political milieus that can strain collaboration. Openly acknowledging that research findings that benefit one group might disadvantage another can help avert distrust. Moreover, fostering shared appreciation of the personal and professional benefits of PH-PBRN participation can strengthen partnerships. The dissemination of results, for example, can be planned such that findings reach the widest range of audiences, assure partner satisfaction, and strengthen PH-PBRN sustainability.

Through this exploration, we recognize that co-learning and capacity-building give practitioners an increased understanding of the research process and give academics a better understanding of practice settings. Additionally, investments in co-learning and capacity-building represent a commitment to the partnership, thereby increasing the likelihood of successful research. Consequently, these investments can be useful in growing PH-PBRN partnerships that can help identify and fill knowledge gaps about public health systems performance and service delivery, ultimately improving the practice of evidence-based public health.

### Implications and Recommendations for Partnerships

While we agree with Israel and colleagues that not all CBPR principles will be applicable to all partnerships (Israel et al., 2003), the reflective process we undertook in this review allowed each of us to consider how the principles might apply directly to or be adapted for PH-PBRN networks both to enhance the collaborative research process and to optimize the real-world impact of findings. We also acknowledge that PH-PBRNs engage communities of practice and exist on a continuum of engagement that depends on the partners’ interests, resources, and availability. Therefore, PH-PBRNs do not always meet the strict parameters defining CBPR.

However, as PH-PBRNs seek to strengthen their partnerships, we recommend that they engage in reflective and inclusive examinations of CBPR principles to consider strategies that best fit the goals and circumstances of their individual partnerships. In particular, we suggest that PH-PBRNs might strengthen their practice by establishing operating norms, principles, and organizational structures reflective of the principles reviewed here and revisit these as their partnerships mature (Cargo & Mercer, 2008; Gust & Seifer, 2011). Coalition or partnership development is context dependent (Kegler, Rigler, & Honeycutt, 2010). Consequently, we recommend that each PH-PBRN examine the CBPR literature, which is replete with tools, examples, and lessons learned for partnership development from which they may benefit (Andrews, Cox, Newman, & Meadows, 2011; Becker, Israel, & Allen, 2005; Fawcett, Schultz, Watson-Thompson, Fox, & Bremby, 2010; Israel, Lichtenstein, et al., 2001; Johnson et al., 2009; KU Work Group for Community Health and Development, 2016; Lewis et al., 2016).

## Figures and Tables

**Table 1.** CBPR Principles Applied to PH-PBRNs.

CBPR principle	Application to PH-PBRNs
1. CBPR recognizes community as a unit of identity	• Community is defined as local and state public health practitioners with shared interest in improving public health practice
2. CBPR involves systems (partnership) development through a cyclical and iterative process	• Build trust among stakeholders through conscious and deliberate attention to understanding each other’s cultural and political milieus • Commit to partnership development and sustainability
3. CBPR facilitates collaborative, equitable partnership in all phases of the research	• Acknowledge power imbalances exist in relationships among PH-PBRN researchers and practitioners • Define and operationalize equity by and for network members • Acknowledge tensions exist regarding control over decision making in the research process, resource sharing, and results dissemination • Pay explicit attention to role assignment, research decision points, compensation, and practitioner-oriented venues for early dissemination
4. CBPR integrates and achieves a balance between research and action for the mutual benefit of all partners	• Generate new knowledge for *organizational* change to inform public health decision • Articulate professional and personal goals for network participation • Promote sensitivity to differing practice and academic cultural and political milieus
5. CBPR promotes co-learning and capacity-building among all partners	• Researchers contribute scientific training; practitioners contribute practice-wisdom and relevance • Co-learning and capacity-building demonstrate a commitment to the partnership and enhance the likelihood of success
6. CBPR builds on strengths and resources within the community	• Acknowledge variation in network composition and interaction patterns • Outreach to practice/academic communities to increase participation, and build skill sets and resources
7. CBPR emphasizes local relevance of public health problems and ecological perspectives that recognize and attend to the multiple determinants of health and disease	• PH-PBRN research focuses on the outer rings of the ecological model
8. CBPR disseminates findings and knowledge gained to all partners and involves all partners in the dissemination process	• Practitioner partners can legitimize research to the practice community in ways that academics cannot • Academics can legitimize practice to the academic community in ways practitioners cannot • Acknowledge and address varying partner preferences for dissemination routes and target audiences
9. CBPR involves a long-term process and commitment	• Emphasize partnership sustainability when resources are scarce • Routine interactions between public health practitioners and academics provide the foundation for partnership development and maintenance

*Note.* CBPR = community-based participatory research; PH = public health; PBRN = practice-based research networks.
